# The role of the aging microenvironment on the fate of PDGFRβ lineage cells in skeletal muscle repair

**DOI:** 10.1186/s13287-022-03072-y

**Published:** 2022-08-05

**Authors:** Aiping Lu, Chieh Tseng, Ping Guo, Zhanguo Gao, Kaitlyn E. Whitney, Mikhail G. Kolonin, Johnny Huard

**Affiliations:** 1 Center for Regenerative and Personalized Medicine, Steadman Philippon Research Institute, 181 West Meadow Drive, Suite 1000, Vail, CO 81657 USA; 2 M.D. Anderson Cancer Center, The University of Texas Health Science Center, Houston, TX 77030 USA; 3 Institute of Molecular Medicine, The University of Texas Health Science Center, Houston, TX 77030 USA

**Keywords:** Mesenchymal stem cells, PDGFRβ lineage cells, Aging, Muscle progenitor cell, Skeletal muscle injury, Fibrosis and fatty infiltration

## Abstract

**Background:**

During aging, perturbation of muscle progenitor cell (MPC) constituents leads to progressive loss of muscle mass and accumulation of adipose and fibrotic tissue. Mesenchymal stem cells (MSCs) give rise to adipocytes and fibroblasts that accumulate in injured and pathological skeletal muscle through constitutive activation of platelet-derived growth factor receptors (PDGFRs). Although the role of the PDGFRα has been widely explored, there is a paucity of evidence demonstrating the role of PDGFRβ in aged skeletal muscle.

**Methods:**

In this study, we investigated the role of PDGFRβ lineage cells in skeletal muscle during aging by using Cre/loxP lineage tracing technology. The PDGFR-Cre mice were crossed with global double-fluorescent Cre reporter mice (mTmG) that indelibly marks PDGFRβ lineage cells. Those cells were analyzed and compared at different ages in the skeletal muscle of the mice.

**Results:**

Our results demonstrated that PDGFRβ lineage cells isolated from the muscles of young mice are MPC-like cells that exhibited satellite cell morphology, expressed Pax7, and undergo myogenic differentiation producing myosin heavy chain expressing myotubes. Conversely, the PDGFRβ lineage cells isolated from muscles of old mice displayed MSC morphology with a reduced myogenic differentiation potential while expressing adipogenic and fibrotic differentiation markers. PDGFRβ lineage cells also gave rise to newly regenerated muscle fibers in young mice after muscle injury, but their muscle regenerative process is reduced in old mice.

**Conclusions:**

Our data suggest that PDGFRβ lineage cells function as MPCs in young mice, while the same PDGFRβ lineage cells from old mice undergo a fate switch participating in adipose and fibrotic tissue infiltration in aged muscle. The inhibition of fate-switching in PDGFRβ lineage cells may represent a potential approach to prevent fibrosis and fatty infiltration in skeletal muscle during the aging process.

## Background

Skeletal muscle is a highly dynamic tissue with remarkable regenerative capacity [1]. Muscle progenitor cells (MPCs) are primarily responsible for skeletal muscle repair [2–4], while satellite cells (SCs), a major source of MPCs, serve a central role in postnatal maintenance, growth, repair, and regeneration in adult muscle [5, 6]. In healthy muscles, SCs are maintained in a quiescent state, lying beneath the basal lamina surrounding each myofiber that promptly activate in response to muscle injury to re-establish homeostatic functions [4]. There are many other MPC populations with myogenic potential have been characterized through various approaches [7–12]. Our group has reported a preplate technique to isolate MPCs based on their adhesion characteristics [13, 14]. Muscle regeneration is a complex phenomenon that requires a close collaboration between MPCs and other muscle resident cell populations, such as mesenchymal stem cells (MSCs), fibro-adipogenic progenitors (FAPs), mesoangioblasts, pericytes, and immune cells [15–18]. During aging, the regenerative capacity of MPCs decline, and perturbations of MPC function and their constituents lead to progressive loss in muscle mass accompanied by the accumulation adipose and fibrotic scar tissue [19, 20]. FAPs have recently emerged as important player of muscle regeneration by interacting with MPCs to promote the formation of new muscle fibers [19, 21, 22]. Newer evidence has demonstrated that FAPs do not generate myofibers but enhance the myogenic differentiation of MPCs after acute injury [22]. In fact, transplantation of purified FAPs in a mouse model result in the generation of ectopic white adipose tissue [22] instead of myofibers, suggesting that the microenvironment plays a major determinant in the fate of FAPs. Fatty infiltrates and fibrosis undermine the quality of regenerated muscles and underlie defective regeneration in aging and models of degenerative disease, such as Duchenne muscular dystrophy (DMD) [23]. FAPs are interstitial mesenchymal progenitors expressing platelet-derived growth factor receptors (PDGFRs) [21, 24–28]. Specifically, PDGFRα-positive FAPs has been shown to mediate intramuscular fibrogenesis and adipogenesis [24, 29]. Constitutive activation of PDGFRα signaling is directly linked to the development of fibrosis in skeletal muscle tissue [30], indicating the functional importance of this PDGF receptor. In contrast, the role of the other PDGF receptor expressing cells especially PDGFRβ cells, in aging and diseased skeletal muscle, has not been fully explored.

PDGF signaling is essential for vascular development and primarily responsible for activating PDGFRβ during embryogenesis [31, 32]. PDGFRβ is highly expressed in pericytes and it is use as a marker for pericytes [33, 34]. Pericytes are mural cells surrounding blood vessels, adjacent to endothelial cells, and play a critical role in maturation and maintenance of vascular branching morphogenesis [35]. In vitro studies in cultured endothelial cells indicated that PDGF signaling in PDGFRβ-expressing pericytes stabilizes nascent blood vessels [36]. Indeed, blockade of PDGF/PDGFRβ signaling reduces the number of pericytes and vascular smooth muscle cells, and thereby compromises the integrity and/or functionality of the vasculature in various organs, including the brain, heart, kidney, skin, and eye [37]. It has been reported that in skeletal muscle pericytes contribute to muscle growth through their effects on the SC pool [38–40]. Specifically, PDGFRβ + pericytes promote myogenic differentiation through insulin growth factor-1 (IGF-1) and controlling quiescence through Ang-1 [41]. Ablation of muscle pericytes in mice lead to myofiber hypotrophy and to impaired establishment of stem cell quiescence [41]. These observations are clinically relevant, as muscle degeneration and regeneration are linked to the pericyte population status in humans [42]. While these data indicate that pericytes play a critical role in skeletal muscle regeneration [43], dysfunctional pericytes may drive degenerative processes in muscle [42]. Under controlled culture conditions pericytes have been shown to differentiate into adipocytes [38]. It has also been reported that PDGFRβ + cells contribute to skeletal muscle and cardiac fibrosis [44] in response to injury [45]. The possibility that the multifaceted roles of PDGFRβ + lineage cells in skeletal muscle could change during aging has not been extensively studied.

In this study, we hypothesized that PDGFRβ + lineage cells directly contribute to muscle regeneration in young mice after injury and play an important role in adipose infiltration and fibrosis development in skeletal muscle of aged mice. To test this hypothesis, we utilized a lineage tracing approach, based on the Cre/loxP technology, which indelibly marks PDGFRβ lineage cells. The PDGFRβ-Cre mice were crossed with ROSA^mT/mG^ mice which is a cell membrane-targeted, double-fluorescent system allows direct visualization of PDGFRβ lineage. Our data suggested that PDGFRβ lineage cells function as MPCs and regenerate skeletal muscle in young mice, while the same PDGFRβ lineage cells in aged mice undergo a fate switch participating in adipose and fibrotic tissue infiltration in skeletal muscle.

## Materials and methods

### Animals

PDGFRβ-Cre;mTmG mice were generated by crossing PDGFRβ-Cre and Rosa^mTmG^ mice (Jackson laboratory) as reported [46]. All animal protocols used for all the experiments were approved by the University of Texas’s Animal Care and Use Committee. The methods were performed in accordance with the approved guidelines and regulations.

### Muscle progenitor cell isolation and culture

We used a previously reported MPC isolation preplate technique based on differential adhesion [13, 14]. Briefly, skeletal muscle tissue was minced and processed through a series of enzymatic dissociations, including 0.2% of collagenase type XI (Sigma-Aldrich, C7657) for 1 h, 2.4 units/ml of dispase (Invitrogen, 17105-04) for 45 min, and 0.1% of trypsin–EDTA (Invitrogen, 15400-054) for 30 min at 37 °C. After enzymatic dissociation, the muscle cells were centrifuged and resuspended in proliferation medium (Dulbecco’s modified Eagle’s medium [DMEM], Invitrogen, 11995-073) supplemented with 10% fetal bovine serum (FBS, Invitrogen, 10437-028), 10% horse serum (HS, Invitrogen, 26050-088), 0.5% chicken embryo extract (CEE, Accurate Chemical Co, CE650T-10), and 1% penicillin–streptomycin (Invitrogen, 15140-122). The muscle cells were then plated on collagen type I (Sigma-Aldrich, C9791) coated flasks. Different populations of muscle-derived cells were isolated based on their adhesion characteristics [13]. The muscle cells have been identified and classified based on their morphology, cell surface markers, and functionality [47–49]. Rapidly adhering cell populations contain mostly MSCs/fibroblasts (preplate population 1 and 2; PP1-2) and satellite cell-derived myoblasts (PP3-5), the slow-adhering cells (PP6) have previously been described to contain the MPC fraction [13]. Those cell populations were obtained and cultured in proliferation medium for further analyze.

### Myogenic differentiation assay and fast myosin heavy chain staining

The muscle cells were plated on 24 well plates (30,000 cells/well) in DMEM supplemented with 2% FBS to promote myogenic differentiation (myotube formation). Three days after plating, immunocytochemical staining for fast myosin heavy chain (MyHCf) was performed. After rinsing two times with PBS, cells were fixed for 5 min in cold methanol (− 20 °C), blocked with 10% donkey serum (017-000-121, Jackson ImmunoResearch) for 1 h, and then incubated with a mouse anti-MyHCf (M4276, 1:250, Sigma-Aldrich, St. Louis, MO, USA) antibody for 2 h at RT. The primary antibody was detected with an Alexa 594-conjugated anti-mouse IgG antibody (A21203, 1:500, Invitrogen, Waltham, MA, USA) for 30 min. The nuclei were revealed by 4, 6-diamidino-2-phenylindole (DAPI, D9542, 100 ng/ml, Sigma-Aldrich, St. Louis, MO, USA) staining. The percentage of differentiated myotubes was quantified by the number of nuclei in MyHCf positive myotubes relative to the total number of nuclei. All stained cells were visualized on a Nikon Eclipse fluorescence microscope.

### Muscle injury procedure

Approximately, 30 µl of 4 µM of CTX (C9759, Sigma-Aldrich, St. Louis, MO, USA) dissolved in PBS was injected into the gastrocnemius muscles (GM) of 5-week, and 12-month and 22-month-old PDGFRβ-Cre;mTmG mice. Five days after injury, the mice were sacrificed, and the muscles were harvested. Muscle tissues were flash frozen in liquid nitrogen-cooled 2-methylbutane and cryosectioned at 10 µm in thickness.

### RNA isolation and qRT-PCR assay

Total RNA from sorted GFP + cells was isolated using TRizol Reagent (Invitrogen, Waltham, MA, USA) and reverse transcribed using the iScript reverse transcription supermix cDNA synthesis kit (Bio-Rad, Hercules, CA, USA) according to the manufacturer’s protocol. Real-time PCR (qRT-PCR) was carried out using the Applied Biosystems™ SYBR™ Green Assay kit (Thermo Fisher, Winsor, NJ, USA) and an Applied Biosystems StepOnePlus RT-PCR thermocycler (Applied Biosystems, San Francisco, CA, USA). Primers were designed using PRIMER-Blast (NCBI) and their sequence has been described in Table 1.

**Table 1:** Primer sequences

Gene	Primer sequence (5′–3′)
GAPDH	Forward: TCCATGACAACTTTGGCATTG Reverse: TCACGCCACAGCTTTCCA
PDGFRα	Forward: TGGCATGATGGTCGATTCTA Reverse: CGCTGAGGTGGTAGAAGGAG
PDGFRβ	Forward: CCGGAACAAACACACCTTCT Reverse: TATCCATGTAGCCACCGTCA
Pax3	Forward: ACCCAAGCAGGTGACAACG Reverse: CTAGATCCGCCTCCTCCTCT
Collagen I	Forward: TCATCGTGGCTTCTCTGGTC Reverse: GACCGTTGAGTCCGTCTTTG
PPARγ	Forward: TTGCTGAACGTAAGCCCATCGAGG Reverse: GTCCTTGTAGATCTCCTGGAGCAG
Pax7	Forward: GTGCCCTCAGTGAGTTCGAT Reverse: CCACATCTGAGCCCTCATCC

### Immunohistochemical analyses in vitro and in vivo

Skeletal muscle cryosections and culture cells were fixed in 5% formalin or 4% paraformaldehyde for 10 min and pre-incubated in 10% donkey serum (017-000-121, Jackson ImmunoResearch) in PBS for 1 h at room temperature (RT). The cryosections and cells were incubated for overnight at 4 °C with primary antibodies for GFP (ab290,1:500, Abcam, Cambridge, UK), PDGFRα (AF1062, 1:200, R&D, Minneapolis, MN, USA), PDGFRβ (ab32570, 1:100, Abcam), collagen type I (SAB4500362, 1:100, Sigma), washed in PBS, and then incubated for 30 min at RT with secondary antibodies: Alexa 488-conjugated donkey anti-rabbit IgG (A21206, 1:500, Invitrogen), Biotinylated anti-Rabbit IgG (BA-1000, 1:300, Vector), Biotinylated anti-goat IgG (BA-5000, 1:300, Vector), Cy5-streptavidin (434316, 1:500, ThermoFisher) was added to act as the tertiary antibody. A Mouse on Mouse kit (BMK-2202, Vector, Olean, NY, USA) was used for Pax7 (DSHB, 1:100) and eMyHC (DSHB, F1.652C,1:50) staining according to the manufacturer’s protocol. Cy3-streptavidin (1:500, GEPA43001, Sigma-Aldrich) was added to act as the tertiary antibody. The nuclei were revealed by 4, 6-diamidino-2-phenylindole (DAPI, D9542, 100 ng/ml, Sigma-Aldrich) staining. All stained sections and cells were visualized on a Nikon Eclipse E800 fluorescence microscope. Six images were randomly selected per slide and GFP + cells, as well as the GFP+/eMyHC + fibers, were manually counted based on 20 × images.

### Fluorescence-activated cell sorting

Fluorescence-activated cell sorting (FACS) (FACSAria II, BD, Franklin Lakes, NJ, USA) was used to isolate mG + (green) cells from freshly digested skeletal muscle tissues of young and old PDGFRβ;mTmG mice. Cells were cultured in proliferation medium.

### Statistical analysis

All results are given as the mean ± standard deviation (SD). Means from young and old mice were compared using the student’s *t* test. Differences were considered statistically significant when *p* < 0.05.

## Results

### PDGFRβ lineage cell contribution to myofibers declines with age

**Fig. 1:**
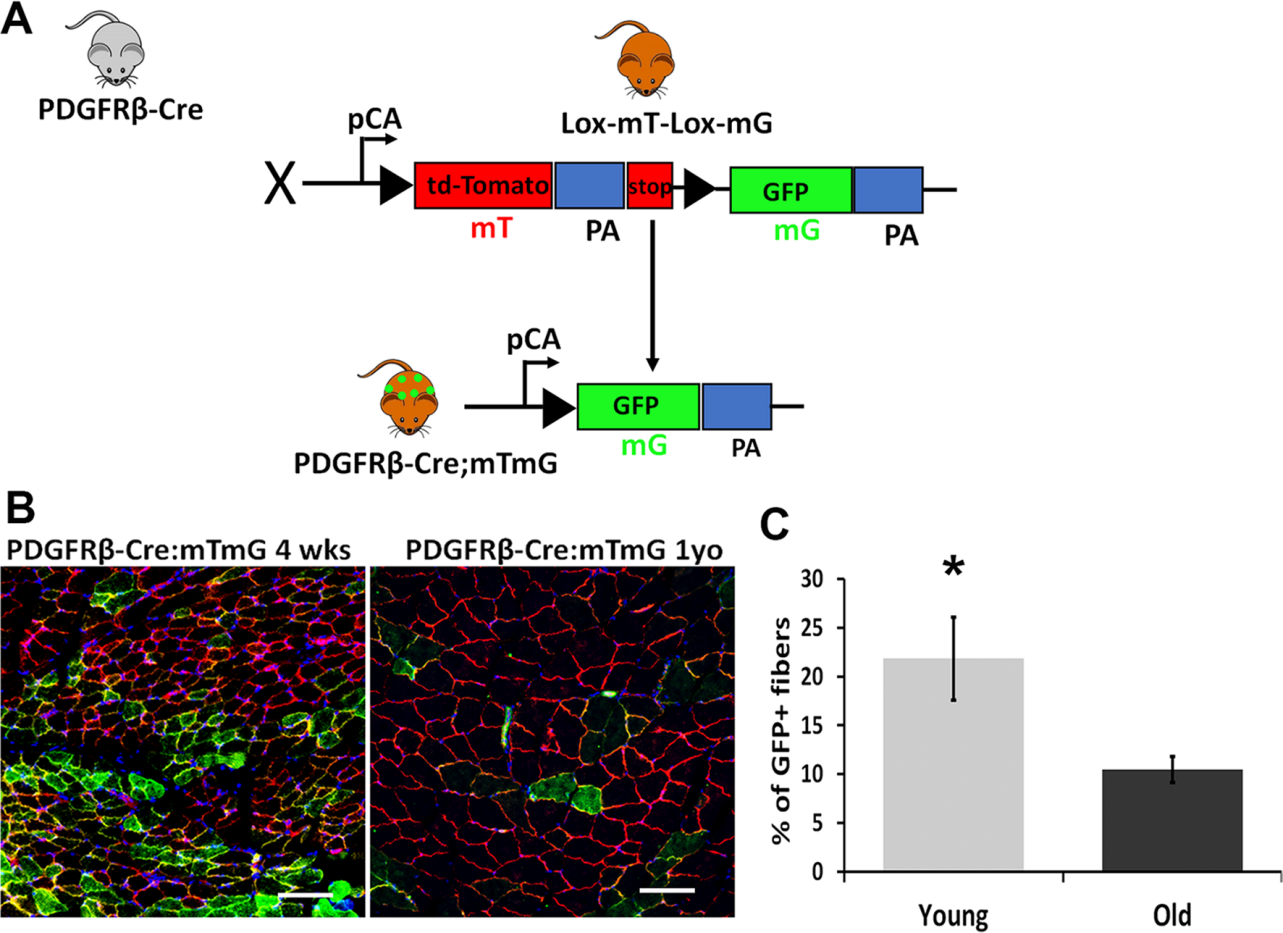
PDGFRβ lineage cells contribute to myofibers in skeletal muscle of young mice. **A** Schematic diagram of crossing PDGFRβ-Cre with mTmG mice. **B** Images representing GFP immunofluorescence staining of GM in 4-week-old and 12-month-old mice. Arrows: myofibers, arrowheads: perivascular cells. **C** Graph shows quantitative mG + myofiber analysis in GM from young and old mice (*n* = 6). Nuclei stained blue (DAPI). Error bars indicating ‘mean ± SD,’ *p < 0.05. Scale bars = 50 µm

To investigate the role of PDGFRβ lineage cells during aging, the PDGFRβ-Cre mice were crossed with Rosa^mTmG^ mice, a two-color fluorescent Cre-reporter strain. When the ROSA^mT/mG^ were bred to PDGFRβ-Cre recombinase expressing mice, the resulting offspring have the gene for membrane-targeted RFP (mT) deleted in the cre-expressing cells, which switch to expressing membrane-targeted EGFP (mG). This allows tracing of the PDGFRβ lineage cells in the PDGFRβ-Cre;mTmG mice as mG + (Fig. 1A). Gastrocnemius muscle (GM) from 4-week-old and 12-month-old PDGFRβ-Cre;mTmG mice was cryosectioned and stained with DAPI. Consistent with what has been reported previously for adipose tissue [46], perivascular GFP + cells were observed in the interstitial muscle space of both young and old mice (Fig. 1B, C). Interestingly, we also observed GFP + myofibers, frequency of which was significantly higher in young mice (Fig. 1B, C, *p* < 0.05). These results show that the PDGFRβ + cell contribution to myogenic lineage declines with age, indicating that PDGFRβ + cells in the muscle of young and old mice may have different roles.

### PDGFRβ lineage cells isolated from young mice are MPC-like cells

**Fig. 2:**
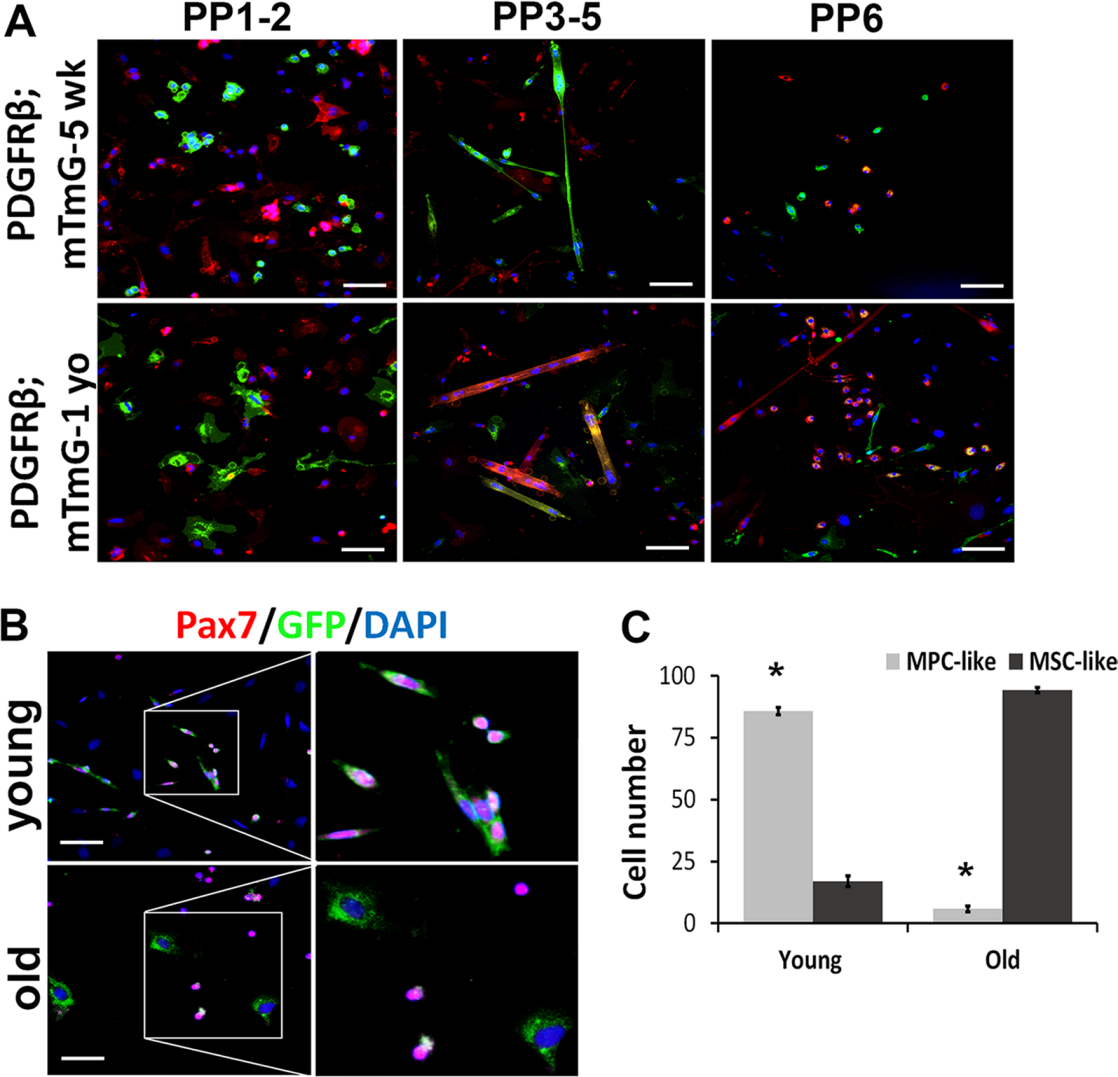
PDGFRβ lineage cells from young and old mice have distinct properties. **A** Images representing the cell morphology of the mT + and mG + cell populations isolated from 5 weeks and 1-year-old mice. **B** IF staining for Pax7 on mG + muscle cells isolated from 5 weeks and 12-month-old mice. **C** Quantification of MPC-like and MSC-like cells in young and old skeletal muscles (*n* = 6). Error bars indicate ‘mean ± SD,’ *p < 0.05. Scale bars in panel *A* = 50 µm, panel *B* = 25 µm

Next, we isolated cells from GM of 4–5-week-old and 12-month-old PDGFRβ-Cre;mTmG mice using a modified preplate technique [14]. In the preplate population 1–2 (PP1-2, early adhered cells), we found that many GFP + cells in the young muscle (Fig. 2A) exhibited SC morphology and expressed Pax7 (Fig. 2B). In contrast, virtually all GFP + cells from GM of old mice displayed morphology typical of MSCs (Fig. 2A) and more importantly did not express Pax7 (Fig. 2B). In the preplate population 3–5 (PP3-5, myoblasts), the majority of myotubes were found to be GFP + in young cell populations, suggesting that the green myotubes fused from PDGFRβ lineage cells (Fig. 2A). In contrast, only occasional GFP + myotubes were found in the cell populations isolated from old PDGFRβ-Cre;mTmG mice (Fig. 2A). In the preplate population 6 (PP6, slow adhered cells, MPCs), we observed that GFP + cells were small, rounded, and exhibited MPC morphology in young muscle, while most GFP + cells from the old muscle displayed fibroblastic morphology (Fig. 2A). In summary, there were significantly more MPC-like GFP + cells in the young mice compared to the old mice, and more MSC like GFP + cells in old mice compared to young mice (Fig. 2C, *p* < 0.05). These results suggest that muscle PDGFRβ lineage cells undergo fate-switching and lose their myogenic potential with age.

### Myogenic differentiation capacity of PDGFRβ lineage cells decreases with age

**Fig. 3:**
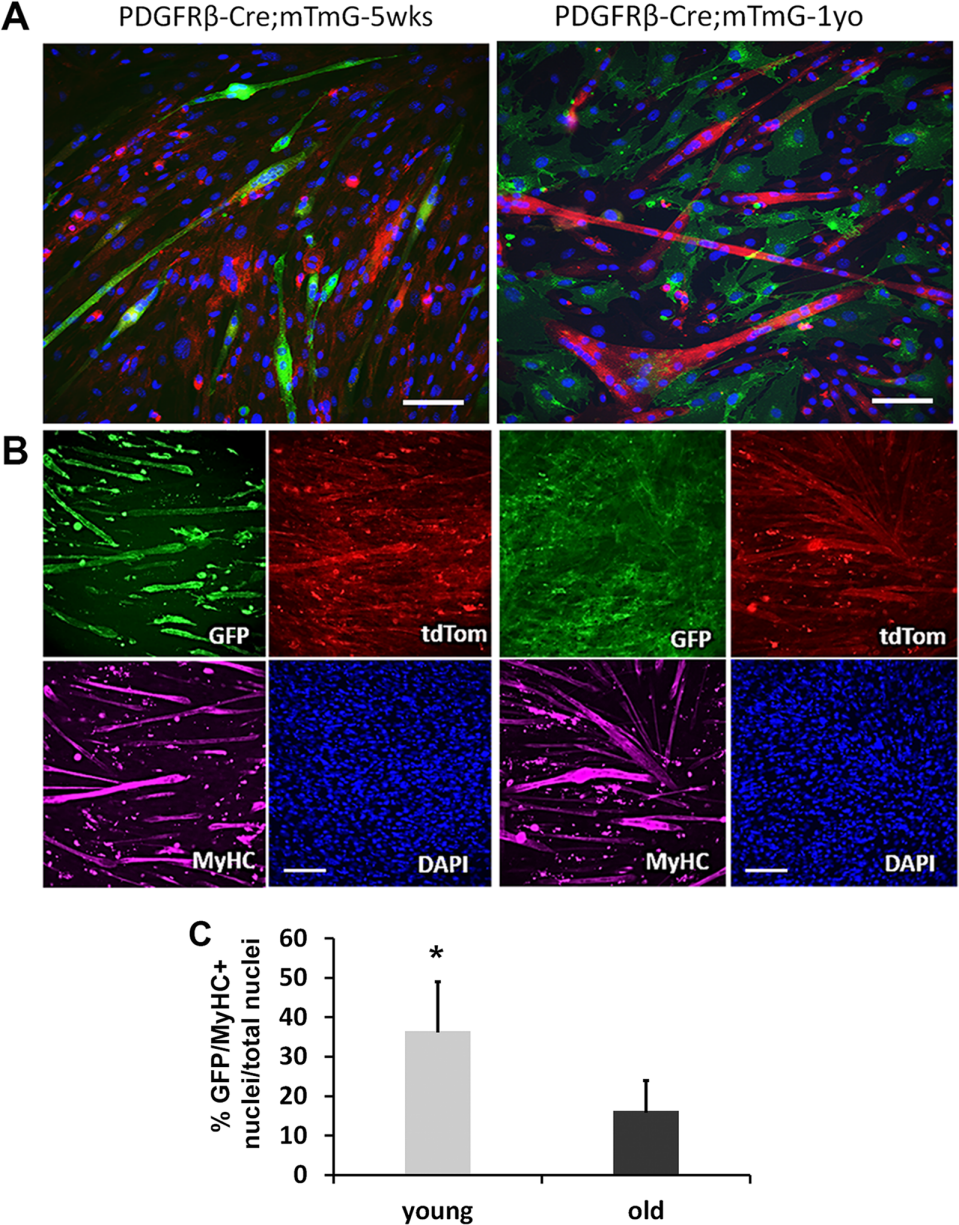
Higher myogenic capacity of PDGFRβ lineage cells from young mice. **A** Myotube formation of MPCs isolated from young and old mice. **B** Myotube formation of myoblasts was assessed by immunofluorescence staining of MyHCf. Staining (purple) shows GFP + myotubes (green) were MyHCf + in young mice, and MyHC + myotubes in old mice were GFP-. Nuclei were stained with DAPI. **C** Quantification of MyHC-f and GFP both positive myotubes (*n* = 6). The percentage of differentiated myotubes was quantified as the number of nuclei in MyHC-f positive myotubes relative to the total number of nuclei. Error bars indicate ‘mean ± SD,’ *p < 0.05. Scale bars = 50 µm

Next, we sought to determine the effect of age on myogenic differentiation of PDGFRβ lineage cells. We subjected MPCs from young and old PDGFRβ-Cre;mTmG mouse GM to myogenic differentiation in vitro. Cells were cultured in proliferation medium to 80% confluence and then switched to myogenic differentiation medium (2% FBS). Three days later, immunofluorescence (IF) staining for MyHCf (a marker of terminal myogenic differentiation) was performed. Notably, many cells from young PDGFRβ-Cre;mTmG mice formed GFP + myotubes (Fig. 3A), indicating that the PDGFRβ lineage cells isolated from young mice retain their high myogenic potential capacity. In contrast, there were relatively more RFP + myotubes in cultures from old PDGFRβ-Cre;mTmG mice (Fig. 3A). These results indicate that PDGFRβ lineage cells lose their myogenic differentiation capacity during aging. Similar results were obtained from the myoblast cell population. The myoblasts isolated from young muscle fused to form MyHCf + GFP + myotubes, while myotubes from aged muscle were primarily MyHCf+GFP− (Fig. 3B). Data quantification confirmed that young PDGFRβ lineage cells have significantly higher myogenic differentiation capacities than the old PDGFRβ lineage cells (Fig. 3C, *p* < 0.05). These results demonstrate that PDGFRβ lineage cells lose their myogenic differentiation capacity during aging.

### PDGFRβ lineage cells isolated from old mice express adipogenic and fibrotic markers

**Fig. 4:**
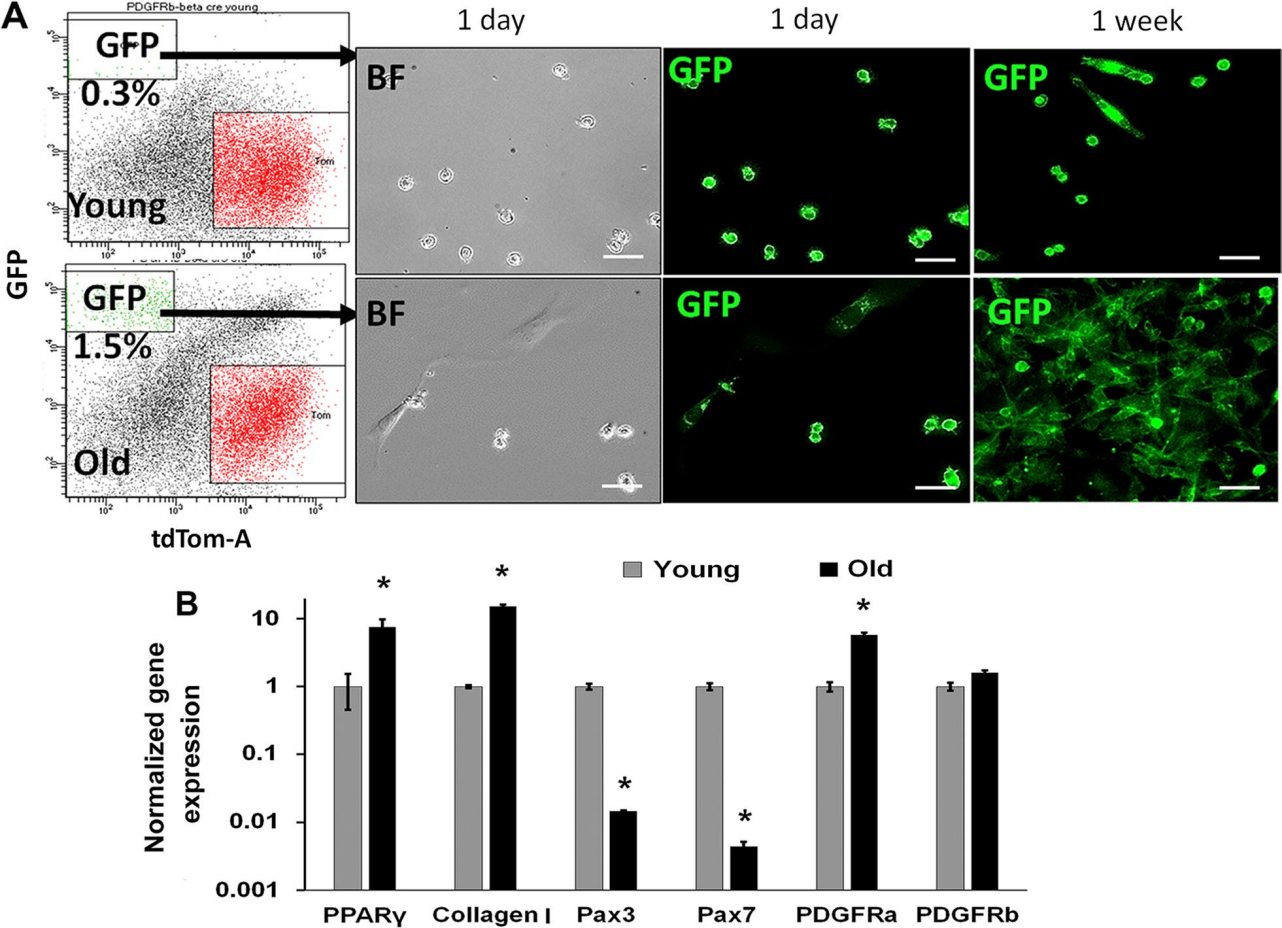
PDGFRβ lineage cells from old mice express fibrotic markers. **A** FACS sorting of the proportion of viable GFP + cells among the total number of cells obtained from skeletal muscle, and images representing the morphology of sorted GFP + cells from young and old mice. **B** qRT-PCR gene expression of myogenic (Pax3, Pax7) and mesenchymal (PDGFRα, PDGFRβ, and collagen type I) cell markers of muscle cells isolated from young and old mice. *N* = 6, error bars indicate ‘mean + SD’, *p < 0.05. Scale bars = 50 µm

To further investigate the purified PDGFRβ lineage cells, we FACS-sorted the GFP + cells (negative for leukocyte marker CD45) from GM of young and old PDGFRβ-Cre;mTmG mice. Interestingly, we found a higher frequency of GFP + cells in muscle of old mice (1.5% ± 0.5) compared to young mice (0.3% ± 0.2) (Fig. 4A). A clear morphologic difference was observed between the young and old GFP+, with many cells from old GM being fibroblastic (Fig. 4A). In addition, after culture for 7 days it was observed that GFP + cells from the old muscles had increased proliferative capacity relative to the GFP + cells from young muscle. As revealed by RT-qPCR, expression of PDGFRβ in GFP + cells from young and old mice were similar (Fig. 4B). A significantly lower expression of myogenic markers (Pax3, Pax7) in GFP + cells from old mice was observed (Fig. 4B, *p* < 0.05). To assess the fate change of PDGFRβ lineage, we chose as markers PPARγ, a master regulator for adipogenesis [50], as well as PDGFRα and collagen type I as markers for fibrosis (22) [51]. Expression of both adipogenic and fibrotic markers was significantly higher in GFP + cells from old mice (Fig. 4B, *p* < 0.05), when compared to their counterpart isolated from young mice. These results suggest that PDGFRβ lineage cells in muscle undergo a fate switch from myogenesis to adipogenesis and fibrosis during the aging process.

### PDGFRβ lineage cells have reduced contribution to muscle regeneration in old mice

**Fig. 5:**
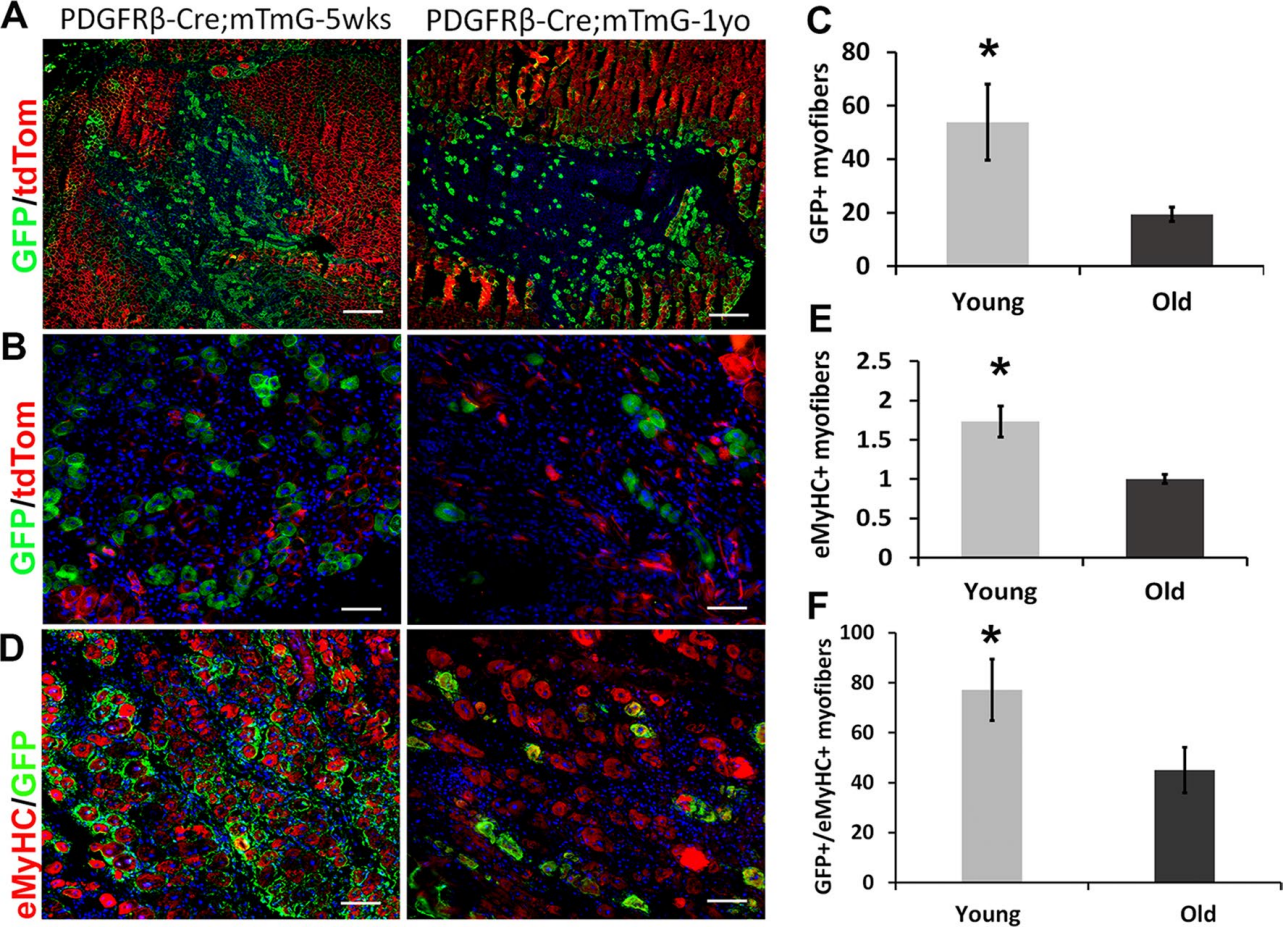
Muscle regeneration capacity of PDGFRβ lineage cells decrease during aging. **A**, **B** Images representing GFP + cells centrally nucleated muscle fibers in young and old muscles 5 days after CTX injury. The images in (**B**) are higher magnification of the images in (**A**). **C** Quantification of regenerated GFP + muscle fibers in skeletal muscle of young and old mice after injury. **D** Images representing GFP and eMyHC staining. **E** Quantification of eMyHC + muscle fibers. **F** Quantification of both GFP + and eMyHC + muscle fibers. *N* = 6, error bars indicate ‘mean + SD’, *p < 0.05. In panel (**A**), all scale bars = 200 µm; in panel (**B**) and (**D**), all scale bars = 50 µm

To test the changes of muscle regeneration potential of PDGFRβ lineage cells with age in vivo, a cardiotoxin (CTX) injury of the GMs was performed on the young and old PDGFRβ-Cre;mTmG mice. We observed significantly more regenerated GFP + myofibers in young mice compared to old mice (Fig. 5A, C, *p* < 0.05). These newly regenerated myofibers were identified based on central nuclear localization (Fig. 5B). We also performed staining for embryonic myosin heavy chain (eMyHC) to confirm the identity of newly regenerated myofibers. As expected, there were significantly more eMyHC + myofibers in young muscle compared to old muscle (Fig. 5E, *p* < 0.05). Importantly, there were more PDGFRβ/GFP + myofibers in young mice co-expressing eMyHC compared to PDGFRβ/GFP + myofibers in old mice (Fig. 5D, F, *p* < 0.05). This result demonstrates that PDGFRβ lineage cells also display a decrease myogenic potential (in vivo) during the aging process.

### PDGFRβ lineage cells in old mice are primarily non-myogenic

**Fig. 6:**
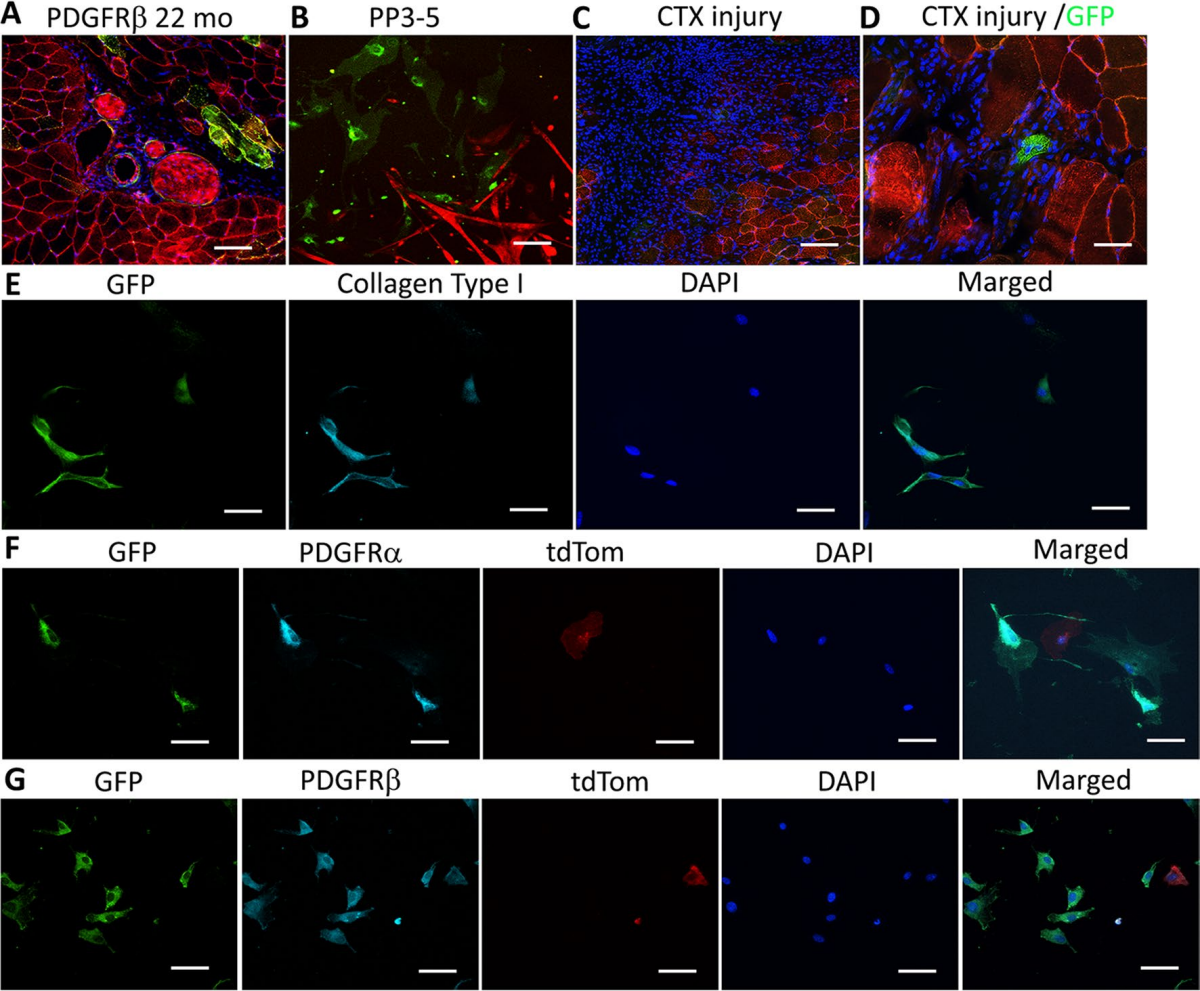
Characterization of PDGFRβ lineage cells in 22-month-old PDGFR-Cre;mTmG mice. **A** GFP immunofluorescence staining of GM of 22-month-old mice. **B** Images of myoblast population isolated from PDGFR-Cre;mTmG mice at 22-month-old. **C** Images of muscle in 22-month-old mice 5 days after CTX injury. **D** Images representing newly regenerated GFP + myofiber after CTX injury. **E** Images of GFP, collagen type I and DAPI staining. **F** Images of GFP, PDGFRα and DAPI staining. **G** Images of GFP, PDGFRβ and DAPI staining. In panels (**A**) and (**C**), scale bars = 50 µm, in panel (**B**), scale bar = 100 µm, all other panels, scale bars = 25 µm

To investigate the fate of PDGFRβ lineage cells during aging progression, we aged the PDGFRβ-Cre;mTmG mice and injured the GM at 22 months of age. First, we found that there were very few GFP + muscle fibers in the muscle of the 22 months old mice (Fig. 6A). Also, we did not observe formation of GFP + myotubes in myoblast cell populations isolated from the muscles of 22-month-old mice (PP3-5, Fig. 6B). Importantly, most of the GFP + cells co-expressed collagen type I (Fig. 6E), PDGFRα (Fig. 6F) and PDGFRβ (Fig. 6G) in 22 months old mice. After cardiotoxin injury in 22-month-old mice, a very low number of GFP + regenerated myofibers was observed (Fig. 6C, D). These results further confirm that PDGFRβ lineage cells lose their myogenic potential (in vitro and in vivo) while acquiring a fibrotic phenotype that contribute to fibrosis in aged skeletal muscle.

## Discussion

During aging, skeletal muscle tissue undergoes progressive muscle wasting and fibrosis [52]. The decline in skeletal muscle regeneration is a hallmark of aging that has been linked to age-related changes on both systemic (sarcopenic signaling) and local (microenvironment) levels [53, 54]. The functional and behavioral changes of muscle stem cells during the aging process remain poorly understood. Several studies that have examined age-related differences in muscle healing at the cellular and molecular levels have found that reduced potency and diminished function of MPCs is a main contributor to muscle catabolism and loss in muscle mass. The progressive decline of muscle regeneration during the aging process has been attributed to a reduced MPC function [43, 55, 56]. Multiple cell types that are present in the skeletal muscle contribute to the maintenance, growth, and regeneration of adult skeletal muscle. Although SCs are generally accepted as a major source of MPCs for adult muscle regeneration, their interaction with other cell populations is necessary for efficient muscle regeneration [57]. The functional heterogeneity of mesenchymal cells within skeletal muscle is increasingly being recognized. PDGFRβ is required for the normal embryonic development of mesenchymal cell types [58]. Various muscle-resident cell types are originated from PDGFRβ lineage, including pericytes and PW1-positive interstitial cells [33, 34, 59]. It is possible that PDGFRβ signaling promotes muscle growth, as both pericytes and PW1-positive interstitial cells can contribute to the myogenic response during regeneration [59]. There has been increasing attention to the multiple roles of PDGFRβ + pericytes during angiogenesis, vascular homeostasis, and pathology [60]. In fact, a recent study showed that pericytes residing in postnatal skeletal muscle differentiate into muscle fibers and generate SCs [39]. A separate study also found that skeletal muscle pericytes are multipotent and exhibit both myogenic and adipogenic potential [14] depending on the environment. Although PDGFRβ + lineage has been investigated in development and regeneration, its role during the aging process has not been fully investigated. Here, we demonstrate that PDGFRβ lineage cells have myogenic potential, express MPC markers, and are able to regenerate muscle fibers after injury in young mice. We show that during the aging process, PDGFRβ lineage cells switch to expressing adipogenic and fibrotic markers while losing their muscle regeneration capacity. Together, our data suggest that PDGFRβ lineage cells serve as MPCs in young mice and undergoes a pro-fibrotic cell fate-switching during aging.

Our group has previously published that cells within the vasculature are at the origin of numerous adult stem cells and that pericytes isolated from a variety of tissues display stem cell characteristics and can be used in cellular therapy to promote muscle repair [8, 14]. Perivascular cells expressing PDGFRβ, CD146, and NG2 (but not hematopoietic, endothelial, and myogenic cell markers) have been characterized in multiple human organs, including skeletal muscle and placenta [61]. In this study, we demonstrate that the PDGFRβ lineage cells can give rise to MPCs, differentiate to myotubes in vitro*,* and regenerate muscle fibers in vivo after injury in young mice. Compared to the SC population, PDGFRβ lineage cells express more perivascular markers, such as CD31, VEGF and CD34 (data not shown), suggesting a distinct cell population.

It has been shown that there are two types of pericytes, (PDGFRα, and PDGFRβ [38]. Although both can participate to muscle regeneration, it is possible that the numbers and function of these different type of pericytes change during the aging process. This could explain the engagement of PDGFRβ lineage into muscle regeneration in young mice while the same cells contribute to fibrosis in old mice.

## Conclusion

Our findings reveal that PDGFRβ lineage cells undergo a fate switch with age, indicating the aging microenvironment may play an important role in the fate of PDGFRβ lineage cells. The inhibition of fate-switching in PDGFRβ lineage cells could be pursued as a potential approach to improving the structure, function, and biology of aged muscle. Future studies will be required to further refine the roles of distinct mesenchymal subpopulations in skeletal muscle repair after injury, disease, and aging.

## Data Availability

All data generated or analyzed during this study are available from the corresponding author on reasonable request.
